# Multidisciplinary approach to the diagnosis of *Contracaecum magnipapillatum* infections in Australian black noddies, *Anous minutus* (Charadriiformes: Laridae)

**DOI:** 10.1007/s00436-023-08050-8

**Published:** 2024-01-10

**Authors:** Shokoofeh Shamsi, Leanne Nelson, Anita Gordon, Kathryn Markham, Nidhish Francis, Jaydipbhai Suthar, Xiaocheng Zhu

**Affiliations:** 1 School of Agricultural, Environmental and Veterinary Sciences, Gulbali Institute, Charles Sturt University, Locked Bag 588, Wagga Wagga, NSW 2678 Australia; 2 Biosecurity Sciences Laboratory, Queensland Department of Agriculture and Fisheries, Archerfield BC QLD, PO Box 156, Brisbane, 4108 Australia; 3 NSW Department of Primary Industries, Wagga Wagga Agricultural Institute, Wagga Wagga, NSW 2650 Australia

**Keywords:** Morphology, Molecular taxonomy, *Anous minutus*, Avian host, Wildlife parasitology

## Abstract

We provide the incidental necropsy findings associated with anisakid nematode infections of black noddy terns, *Anous minutus* Boie, 1844 (Charadriiformes: Laridae), from offshore islands in the southern Great Barrier Reef, Queensland, Australia. Specimens collected from the proventriculi were identified morphologically as *Contracaecum magnipapillatum* Chapin, 1925 (Rhabditida: Anisakidae), using light and scanning electron microscopy (SEM). The entire nuclear ribosomal DNA internal transcribed spacer (ITS) region (ITS1-5.8S-ITS2) was amplified by polymerase chain reaction (PCR) and sequenced to provide reference sequences for morphologically well-identified voucher specimens. Interestingly, after an alignment with closely related taxa using BLAST, sequences of the ITS1 and ITS2 were 100% identical to the sequences assigned to *Contracaecum septentrionale* Kreis, 1955, from a razorbill, *Alca torda* Linnaeus, 1758 (Charadriiformes: Alcidae), from Spain. These results either raise questions about the ITS as a genetic marker for some members of *Contracaecum*, or the identity of the specimens assigned to *C. septentrionale*, given that no supporting morphological data was associated with them. We highlight the need for a combined morphological and molecular approach to parasite diagnostics and the use of multiple genetic loci to resolve the molecular taxonomy of cryptic species. Morphological identifications should be taxonomically robust, transparent and precede the deposition of molecular barcodes in public repositories. The gross and histopathological findings of our investigation concur with previous reports of widespread *Contracaecum* infections in black noddies and support the contention that *Contracaecum* spp. are an unlikely primary cause of mortality.

## Introduction

The black noddy, *Anous minutus*, is a common piscivorous seabird with a tropic and subtropic marine distribution. Vegetated islands off the coast of Queensland, Australia, support a large breeding population (Hill et al. 1997). A series of mass morbidity and mortality events of black noddies along the Queensland coast from mid to late 2021 presented an opportunity to examine the gastrointestinal nematode fauna associated with this ubiquitous seabird. Previous reports of the parasitic nematodes affiliated with this avian host are limited to a few early collections of adult *Contracaecum magnipapillatum* (Syn. *C. magnicollare* Johnson and Mawson, 1941), *Anisakis* sp. (Syn. *Stomachus* sp.) and an unidentified regurgitated *Acuariidae* larva from the Pacific Ocean (Chapin 1925; Johnston and Mawson 1941; 1951; Mawson et al. 1986; Hugot et al. 1991; Fagerholm et al. 1996). The pathological findings associated with infection of black noddies by *C. magnipapillatum* on Heron Island were described by Fagerholm et al. (1996) and remain the only detailed account of parasitism by *Contracaecum* spp. in this host.

This report presents the necropsy findings for three birds infected with *C. magnipapillatum* and provides the first molecular data for the species. We discuss the challenges of resolving the molecular taxonomy of this nematode and its northern hemispheric congener *Contracaecum septentrionale*. We argue for a multidisciplinary approach to the identification of parasites using morphology in combination with molecular techniques.

## Methods

**Fig. 1:**
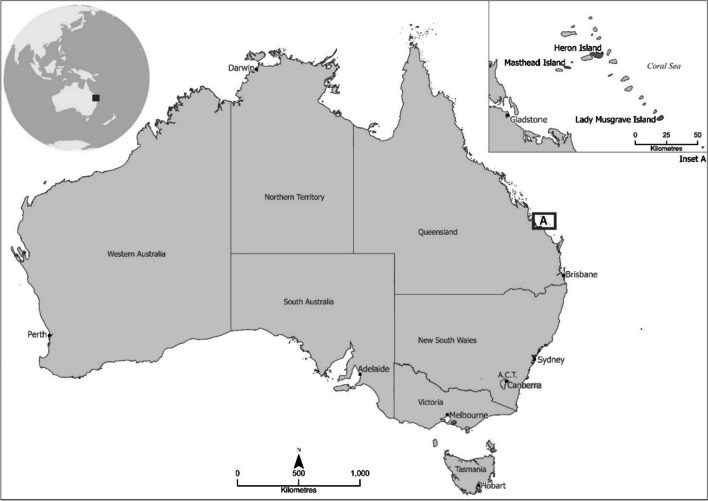
Localities of the black noddies examined in the present study. Inset shows location of islands of interest within the southern Great Barrier Reef

In October 2021, necropsy examinations were performed on black noddies from three offshore islands in the Great Barrier Reef (Fig. 1) as part of a disease investigation into mass morbidity and mortality events at these locations. Birds 1–3 were collected either moribund or freshly dead during October from Masthead, Lady Musgrave and Heron Islands, respectively. Birds 1 and 2 were adult males and bird 3 was a non-breeding adult female. Birds were submitted to the Biosecurity Sciences Laboratory for necropsy examinations. Nematodes were collected from the gastrointestinal tracts of three birds and fixed in 70% ethanol. A subset of 12, 3 and 9 nematode specimens from birds 1–3, respectively, were forwarded to the Shamsi’s Parasitology Laboratory at Charles Sturt University (CSU) for identification.

### Histopathology

Fresh tissue from the proventriculi was dissected and fixed in 10% neutral buffered formalin overnight at room temperature for histological processing. Tissue sections were paraffin-embedded using standard techniques, sectioned at 3 µm and stained with haematoxylin and eosin (HE).

### Morphological examination

Prior to morphological examination of the ethanol-preserved specimens, DNA extractions were carried out by removing a small tissue sample of mid-body from each nematode following the methods described in Shamsi et al. (2016). Specimens were subsequently slide-mounted in lactophenol to clear and examined morphologically using a compound microscope (Olympus CX23, Olympus Corporation, Japan). Nematodes were grouped according to the morphological traits defined in previous studies (Fagerholm et al. 1996; Shamsi et al. 2008; Shamsi et al. 2009a; b): labial structure and morphometry, excretory pore and nerve ring position, oesophageal ventriculus, ventricular appendix, intestinal caecum and the tail. Drawings were made to scale using a microscope drawing tube (BX43 Olympus Microscope, Olympus Corporation, Japan). Voucher specimens were deposited at the Queensland Museum, South Brisbane (reference numbers G240585 – G240586).

### Molecular identification

DNA extractions were performed on subsamples of the mid-body using a DNeasy Blood and Tissue Kit (QIAGEN) according to the manufacturer’s protocol and as modified by Shamsi et al. (2018). The entire ITS region of nuclear ribosomal DNA (ITS1-5.8S-ITS2) was amplified by PCR using primer sets SS1 and NC2 according to the protocols described previously (Shamsi et al. 2019a). PCR products of sufficient strength were sent to the Australian Genome Research Facility (AGRF) for Sanger sequencing. Sequence quality was checked using SeqMan v8.0 (DNASTAR). The consensus sequence was assembled from forward and reverse reads using the BioEdit software v7.2.5 (Hall 1999).

## Results

### Gross and histological findings

Macroscopically, birds 1 and 2 were in poor body condition with no subcutaneous or coelomic fat reserves and marked atrophy of the pectoral muscles. Bird 3 was in moderate to good nutritional condition with obvious subcutaneous and some coelomic fat reserves. In all birds, gastrointestinal content appeared reduced. In birds 1 and 2, approximately 10–20 nematodes were detected in the oesophagus, proventriculus and gizzard; many were associated with focal ulcers in the proventricular mucosa. In bird 3, several nematodes were present in the distal oesophagus and proventriculus where they were associated with a focal nodule of darkened mucosa and brown-black strands of haemorrhage, respectively.

**Fig. 2:**
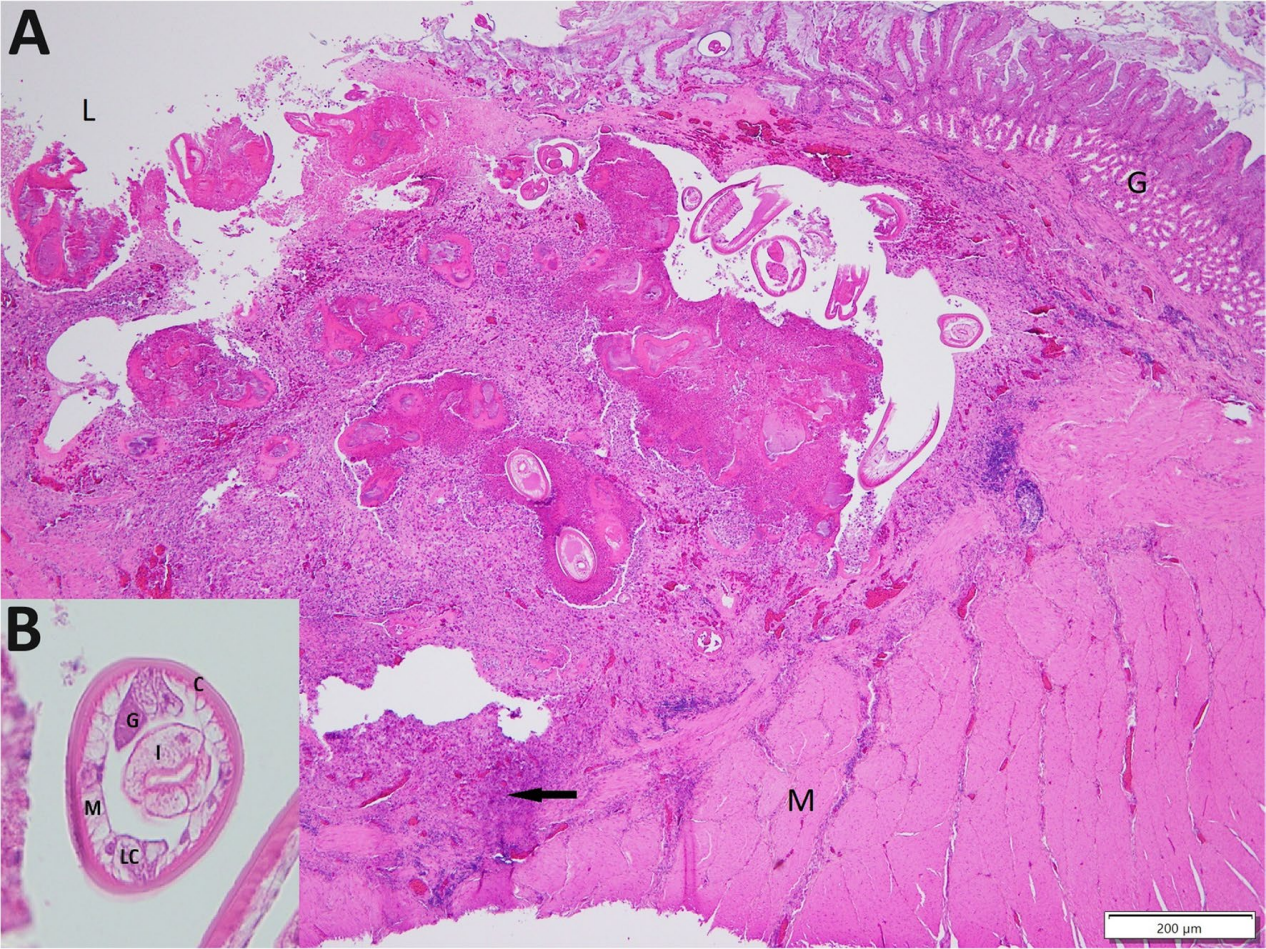
**A** Multiple *Contracaecum* larvae are evident within a deep ulcer of the proventriculus in bird 1. The inflammatory infiltrate (arrow) containing aggregates of bacteria extends to deep into the muscularis layer (M). Glandular mucosa (G), proventricular lumen (L). **B** Higher magnification showing transverse sections of *Contracaecum* larvae. Cuticle (C), intestine (I), lateral chords (LC), gland (G), coelomyarian musculature (M). Note the absence of gonads

Histologically, the focus of ulceration in bird 1 extended from the mucosa well into the submucosal muscle (Fig. 2). Multiple partial longitudinal and cross sections of nematode parasites were present in the affected area, either free within the lumen or embedded in fibrin surrounded by degenerate heterophils in a granulomatous matrix. Nematodes were characterised by a thick cuticle, pseudocoelom, coelomyarian musculature, prominent lateral chords with associated eosinophilic gland cells and an intestine composed of uninucleate columnar cells with a prominent brush border (Fig. 2). Aggregates of bacteria surrounded by fibrin, embedded in eosinophilic cellular debris and degenerate and viable heterophils were present multifocally within the ulcerated area. In bird 3, focal ulceration of the proventriculus was associated with extensive bacterial colonisation, mucosal haemorrhage and chronic, transmural inflammation that included a dense lymphocytic infiltrate in the subserosa. Nematodes were present at the mucosal surface and beneath the koilin layer at the proventricular-gizzard junction. In bird 2, marked autolysis of the proventricular tissue precluded a histological description.

### Morphological identification

**Fig. 3:**
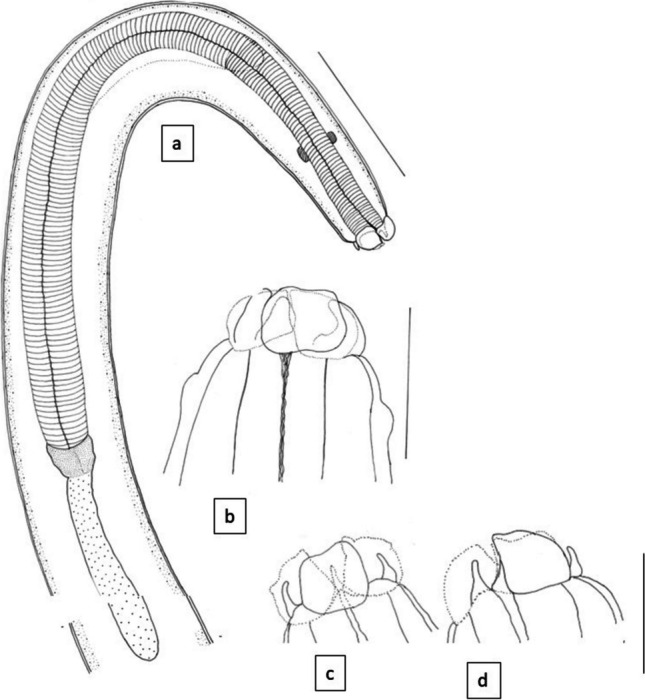
Line drawing of *C. magnipapillatum* from the present study. **a** Anterior end of the parasite. **b**–**d** Labial views

**Fig. 4:**
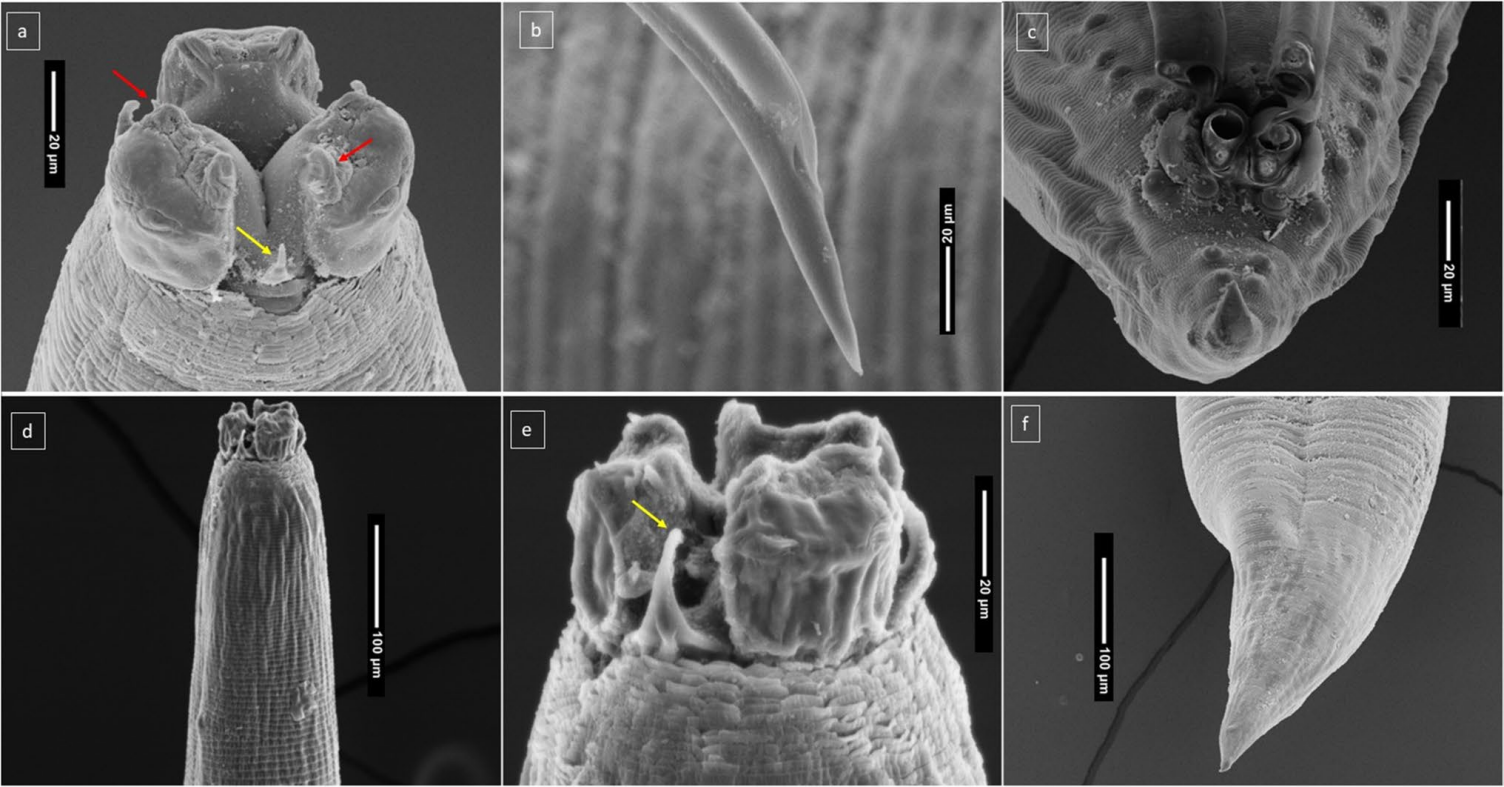
Images of adult *C. magnipapillatum* produced by scanning electron microscopy. **a** Anterior end of male (red arrows indicate hook-like extension of auricle; yellow arrow shows the interlabia). **b** Tip of male spicule. **c** Ventral view of male post-cloacal region. **d** Anterior end of female. **e** Female mouthpart showing ventral labium and one of two subventral interlabia (yellow arrow). **f** Posterior end of female

**Fig. 5:**
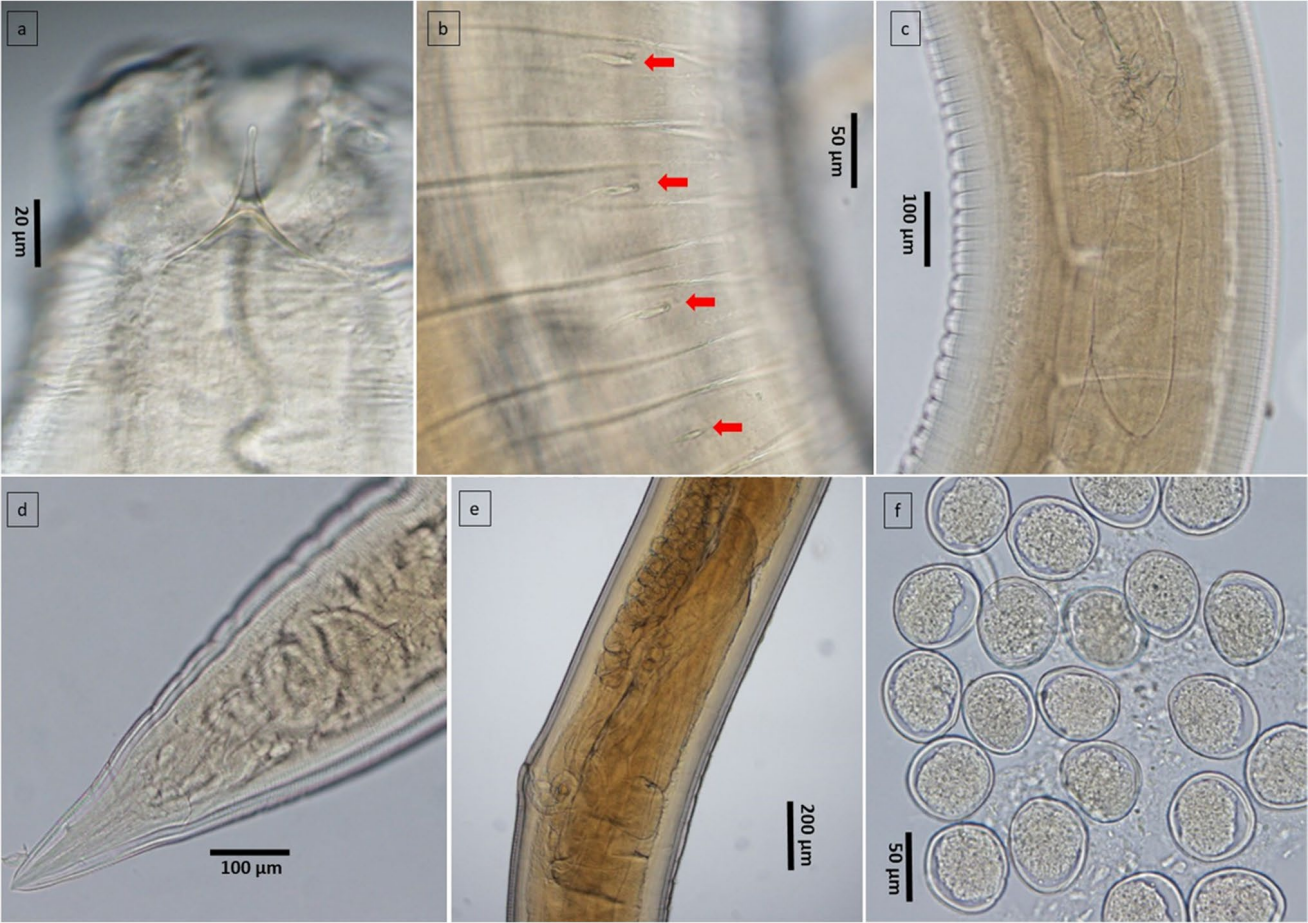
Images of *C. magnipapillatum* using light microscopy. **a** Subventral labium of male. **b** Lateral view of male pre-cloacal papillae (arrows) towards posterior region. **c** Ventriculus and ventricular appendix of male. **d** Posterior end of female. **e** Vulval region of female. **f** Eggs from gravid female

Nematodes were morphologically identified as belonging to the genus *Contracaecum* based on apomorphic characters such as oppositely-directed caecae and anteriorly-located excretory pores. A subset of 5, 3 and 9 nematodes from birds 1 to 3, respectively, were in sufficient morphological condition for a species-level diagnosis using the characters outlined in Table 1. All specimens were identified as adult *C. magnipapillatum* on the basis of general morphometry, genitalia, the finger-like interlabia which lack a distal bifurcation and the labia that possess lateral articles forming inwardly bent hook-like extensions (Figs. 3, 4, and 5).

**Table 1:** Comparison of morphometric characters between specimens of *C. magnipapillatum* from the present study and Fagerholm et al. (1996) and the type specimens of *C. septentrionale* (Kreis 1955). Measurements are presented as ranges in mm unless otherwise stated. Morphometric averages and the number of specimens examined are provided in parentheses

Reference	Present study		Fagerholm et al. 1996		Kreis 1955	
Host	*Anous minutus*		*Anous minutus*		*Phalacrocorax aristotelis*	
Locality	Queensland, Australia (Fig. 1)		Queensland, Australia		Iceland	
Sex	Male	Female	Male	Female	Male	Female
Number examined	9	4	15	10	Unknown	Unknown
Total body length	10.20–14.18 (12.27; *n* = 9)	11.83–17.30 (14.24; *n* = 4)	11.0–19.9	17–26	31.61–38.00	39–45
Body width	0.40–0.60 (0.48; n = 9)	0.43–0.68 (0.56; n = 4)	0.33–0.56	17.8–25.5	0.88–l.24	1.30–l.89
Nerve ring	0.30–0.46 (0.40; *n* = 5)	0.40–0.42 (0.41; 2)	0.40–0.52	0.52–0.57	Not provided	Not provided
Oesophagus	2.00–3.23 (2.49; *n* = 9)	2.24–3.36 (2.80; *n* = 4)	2.23–2.96^?^	2.99–4.14	10.8	9.32
Intestinal caecum	1.42–2.50 (1.79; *n* = 9)	1.67–2.32 (2.02; *n* = 4)	1.52–2.07	2.17–3.52	2.05–2.14	1.51–2.08
Ventricular appendix	0.35–0.54 (0.48; *n* = 7)	0.50–0.75 (0.60; *n* = 4)	0.51–0.67	0.70–1.04 (includes ventriculus length)	0.756–1.029	l.00–1.13
Spicules	2.14–3.63 (2.90; *n* = 8)	Not applicable	2.62–3.65 2.83–3.40	Not applicable	12.57–15.16	Not applicable
No of precloacal papillae	26–36	Not applicable	19–35 (35)	Not applicable	30–35	Not applicable
Vulva	Not applicable	4.83–6.80 from ant end	Not applicable	6.67–8.91	Not applicable	Not provided
Eggs	Not applicable	0.06–0.08 × 0.04–0.06	Not applicable	0.058–0.083* × 0.042–0.063	Not applicable	Not provided
Tail length	0.13–0.19	0.14–0.24	0.13–0.18	0.22–0.27	1.21–1.66	0.9–0.95
Tail width	0.10–0.15	0.10–0.14	Not provided	Not provided	Not provided	Not provided
Oesophagus/body length	0.21 (0.19—0.23)	0.20 (0.19–0.21)	0.14–0.20*	5.65–6.96	0.28–0.34*	0.20–0.23*
Intestinal caecum/oesophagus	0.73 (0.65–0.77)	0.72 (0.69–0.75)	0.56–0.77*	1.24–1.49	0.18–0.19*	0.16–0.22*
Intestinal caecum/ventricular appendix	3.86 (3.17–4.63)	3.43 (2.93–4.64)	Not provided	Not provided	0.35–0.50*	0.48–0.74*

*Not provided in the reference; calculated in the present study

^?^In the original publication, it was noted as 2.23–0.96

### Molecular identification

ITS sequence data were obtained for 5 males and 1 female specimen (bird 1 = 2 specimens, bird 2 = 3 specimens, bird 3 = 1 specimen). All specimens had 100% identical sequences. A BLAST search of the entire ITS region (ITS1-5.8S-ITS2) in GenBank yielded no identical hits, and those with the highest similarity differed to our specimens by at least 53 nucleotides (6%). However, when the ITS1 and ITS2 regions were searched independently from the 5.8S component, they matched sequences assigned to *C. septentrionale* with 100% identity (accession numbers: AJ634784 and AJ634787, respectively). These sequences were obtained from specimens described as “morphologically corresponding to *C. septentrionale*”, but supporting morphological descriptions were not provided. A BLAST search of the highly conserved 5.8S region against members of *Contracaecum* returned percent identities ranging between 97.18 and 98.59% with 100% query cover, but sequences from this locus were not available for *C. magnipapillatum* nor *C. septentrionale*.

## Discussion

Our study is the first to provide both morphological and molecular data for the anisakid nematode species, *Contracaecum magnipapillatum*. Accurate identification of morphologically cryptic parasites, particularly in the context of an expanded disease investigation, should incorporate a multidisciplinary approach utilising traditional morphological techniques such as microscopy (light and SEM), in combination with molecular methods (de Leon and Nadler 2010; Caffara et al. 2023).

To date, *C. magnipapillatum* has been collected from a relatively broad host range almost exclusively in the Pacific (*Anous* spp. and unpublished museum records of piscivorous birds from Procellariidae, Stercorariidae and Sulidae) (Johnston and Mawson 1941; Mawson et al. 1986; Hugot et al. 1991; Fagerholm et al. 1996). Beyond the Pacific, the only published accounts of *C. magnipapillatum* are from a Canadian albatross, *Diomedea* sp. (Mawson 1956) and a great cormorant, *Phalacrocorax carbo* Linnaeus 1758 from Egypt (Al-Bassel 2006). There are several morphological differences that reliably separate *C. septentrionale* from *C. magnipapillatum* including the interlabia and the significantly smaller, non-overlapping body and spicule lengths of the latter (Table 1). *Contracaecum septentrionale* was originally described from the European shag *Phalacrocorax aristotelis* Linnaeus 1761 from Iceland based on male spicule length and morphological features of the anterior end of adults (Kreis 1955). During mass beaching events of the same host in Spain between 1996 and 1997, larvae and adults of *C. septentrionale* were morphologically identified using light microscopy and SEM (Abollo et al. 2001). With the exception where male spicule length was considered (D'Amelio et al. 1990), all other reports of *C. septentrionale* derive from molecular studies where supporting morphological data were not provided (Cianchi et al. 1992; Nadler et al. 2000; Mattiucci et al. 2008; Lin et al. 2013; Mohammad and Hbaiel 2019). Multilocus enzyme electrophoresis (MEE) studies were conducted on specimens assigned to *C. septentrionale* ex. *Phalacrocorax aristotelis* and *P. carbo carbo* from Norway, Iceland, southeastern Canada and the northeast Atlantic (D'Amelio et al. 1990; Cianchi et al. 1992; Mattiucci et al. 2008). A phylogenetic study of *Contracaecum* spp. by Nadler et al. (2000) incorporated a partial nuclear-encoded large-subunit ribosomal DNA sequence (accession no. AF226588) obtained from a single Icelandic specimen of *C. septentrionale* ex *P. carbo* previously identified by isoenzyme analysis from an undisclosed source. Li et al. (2005) sequenced the first and second internal transcribed spacers (ITS1 and ITS2) of specimens morphologically corresponding to *C. septentrionale* collected from *Alca torda* Linnaeus 1758, Spain, and deposited sequences for this species in GenBank (accession numbers: AJ634784 and AJ634787, respectively). More recently, Mohammad and Hbaiel (2019) amplified the ITS1 region of fourth-stage larvae from a black-crowned night heron, *Nycticorax nycticorax* Linneaus 1758 from Iraq and assigned the specimens to *C. septentrionale* because they matched > 99% of the reference sequence for this species (accession no. MK424799) (Li et al. 2005).

The ITS1 and ITS2 of eukaryotes are highly variable nuclear loci widely used as molecular markers to resolve species and phylogenetic relationships among closely related taxa. These markers have been used to distinguish between species within *Contracaecum* and other members of the Anisakidae (e.g. Li et al. 2005; Shamsi et al. 2008; Shamsi et al. 2009b; a; Roca-Geronès et al. 2023) and also investigating the life cycle and transmission patterns of these parasites (e.g. Shamsi et al. 2011, 2017, 2019b). A recent phylogenetic study of the ITS1 and ITS2 regions of *Contracaecum* incorporating sequences obtained from *C. septentrionale* (Li et al. 2005) demonstrated phylogenetically distinct clades among congeners (Roca-Geronès et al. 2023)*.* Given that the ITS1 and ITS2 regions of our specimens morphologically confirmed as *C. magnipapillatum* were 100% identical to those assigned to *C. septentrionale* (Li et al. 2005), we raise the following possibilities: (1): the ITS region is conserved among some members of the *Contracaecum* genus as previously demonstrated for members of *Contracaecum osculatum sensu lato* (Rudolphi, 1802) Baylis, 1920 (Zhu et al. 2000a, 2000b); (2) specimens of *C. magnipapillatum* from the present study and those assigned to *C. septentrionale* from Li et al. (2005), and possibly other cited studies, are the same species (i.e. *C. magnipapillatum*). Avian host range also differs except for the single shared host, *P. carbo*, in the northern hemisphere.

Sequencing additional loci such as the four mitochondrial DNA regions previously shown to discriminate between species of *Contracaecum* (Mattiucci et al. 2008; Lin et al. 2013; Caffara et al. 2023) would provide further insight into the molecular taxonomy of *C. magnipapillatum* and *C. septentrionale*. Mitochondrial sequences assigned to *C. septentrionale* are available for comparative analysis in GenBank (Lin et al. 2013) and were probably obtained from the same cohort of specimens collected from *Alca torda*, Spain by Li et al. (2005). Constraints of the current project and our inability to source suitable additional reference material of both species prevented the pursual of further molecular work. This represents a caveat to this study but provides an opportunity for further review. Future studies should compare multiple genetic loci from morphologically verified collections of *C. septentrionale* and *C. magnipapillatum*, ideally from geographically diverse hosts.

Gross and histopathological findings in the proventriculi of infected birds were consistent with previous descriptions of *Contracaecum* spp. infections in seabirds (Fagerholm et al. 1996; Abollo et al. 2001). *Contracaecum* spp. nematodes were detected at necropsy in about one-third of the black noddies investigated during the mortality event, but the nematodes were only occasionally associated with grossly visible mucosal ulcers. There was no clear association between the presence of nematodes or ulcers and subjective body condition score, with the proventricular lesions seen in some birds considered incidental and not the cause of mortality. Findings in this study support the contention that *Contracaecum* spp. are an unlikely primary cause of mortality in many seabird hosts (Abollo et al. 2001; Fagerholm and Overstreet 2008; Ladds 2009).

In conclusion, our research presents both morphological and molecular information on the anisakid nematode species known as *Contracaecum magnipapillatum*. When it comes to accurately identifying morphologically similar parasites, especially within the context of an extensive disease investigation, it is crucial to adopt a multidisciplinary approach. Published taxonomic keys and original descriptions should be the primary point of reference for verification of specimens. The deposition of voucher specimens in publicly accessible collections should supplement published literature so that researchers can access material for review or further testing.

## Data Availability

Upon request.

## References

[CR1] Abollo E, Gestal C, Pascual S (2001). Anisakid infection in the European shag *Phalacrocorax*
*aristotelis*
*aristotelis*. J Helminthol.

[CR2] Al-Bassel D (2006). Scanning electron microscopic study on *Contracaecum magnipapillatum* (Nematoda: anisakidae) from cormorants in wadi Al-Raiyan Lake area, Fayoum. Egypt. J Egyptian German Soc Zool.

[CR3] Caffara M, Tedesco P, Davidovich N, Locke SA, Gustinelli A, King R, Nuytten M, Nuzzo M, Fioravanti ML (2023). Advancing understanding of the taxonomy and diversity of the genus *Contracaecum* in the great white pelican (*Pelecanus*
*onocrotalus*). Parasitol Res.

[CR4] Chapin EA (1925). Descriptions of new internal parasites. Proc United States Nat Mus.

[CR5] Cianchi R, Orecchia P, Berland B, Paggi L, D’Amelio S, Mattiucci S, Nascetti G, Bullini L (1992) Genetic studies on some Contracaecum species, parasites of fish-eating birds. The Netherlands, Proc VIth Europ Multicolloq Prasitol, The Hague, p 127

[CR6] D’Amelio S, Nascetti G, Mattiucci S, Cianchi R, Orecchia P, Paggi L, Berland B, Bullini L (1990). Ricerche electroforetiche su alcune specie del genere *Contracaecum*, parassiti di uccelli ittiofagi (Ascaridida: Anisakidae). Parassitologia.

[CR7] de Leon GPP, Nadler SA (2010). What We Don't Recognize Can Hurt Us: A Plea for Awareness About Cryptic Species. J Parasitol.

[CR8] Fagerholm HP, Overstreet RM, Humphrey-Smith I (1996). *Contracaecum*
*magnipapillatum* (Nematoda, Ascaridoidea): resurrection and pathogenic effect of a common parasite from the proventriculus of *Anous*
*minutus* from the Great Barrier Reef, with a note on *C*. *variegatum*. Helminthologia.

[CR9] Fagerholm HP, Overstreet RM (2008) Ascaridoid nematodes: *Contracaecum*, *Porrocaecum*, and *Baylisascaris*. In: Atkinson CT, Thomas NJ, Hunter DB (eds) Parasitic diseases of wild birds. Iowa, USA, Wiley-Blackwell, pp 413–433

[CR10] Hall TA (1999). BioEdit: a user-friendly biological sequence alignment editor and analysis program for Windows 95/98/NT. Nuc Acids Symp Ser.

[CR11] Hill GJ, Carter JL, Barnes A, Dyer PK, Rosier D (1997). The Black Noddy breeding population at Heron Island, Great Barrier Reef: 1985–1989. Corella.

[CR12] Hugot JP, Morand S, Vassart M (1991). Morphological study of *Contracaecum*
*magnicollare* (Nematoda : Anisakidae) from *Anous*
*minutus* (Aves, Laridae). Syst Parasitol.

[CR13] Johnston TH, Mawson PM (1941). Ascaroid nematodes from Australian birds. Trans Roy Soc S Aust.

[CR14] Johnston TH, Mawson PM (1951). Report on some parasitic nematodes from the Australian Museum. Rec Aust Museum.

[CR15] Kreis HA (1955). *Contracaecum **septentrionale*, ein neuer parasit aus dem Kormoran. Sein Lebenslauf, sowie Angaben uber die Entwicklung der Anisakinae Z Parasitkde.

[CR16] Ladds P (2009) Pathology of Australian native wildlife. CSIRO Publishing, p 640

[CR17] Li A, D'Amelio S, Paggi L, He F, Gasser RB, Lun Z, Abollo E, Turchetto M, Zhu X (2005). Genetic evidence for the existence of sibling species within *Contracaecum*
*rudolphii* (Hartwich, 1964) and the validity of *Contracaecum*
*septentrionale* (Kreis, 1955) (Nematoda: Anisakidae). Parasitol Res.

[CR18] Lin R-Q, Liu G-H, D'Amelio S, Zhang Y, Song H-Q, Weng Y-B, Zou F-C, Zhu X-Q (2013). Sequence variation in four mitochondrial genes among sibling species within *Contracaecum*
*rudolphii sensu lato*. Mol Cell Probe.

[CR19] Mattiucci S, Paoletti M, Olivero-Verbel J, Baldiris R, Arroyo-Salgado B, Garbin L, Navone G, Nascetti G (2008). *Contracaecum*
*bioccai* n. sp. from the brown pelican *Pelecanus*
*occidentalis* (L.) in Colombia (Nematoda: Anisakidae): morphology, molecular evidence and its genetic relationship with congeners from fish-eating birds. Syst Parasitol.

[CR20] Mawson PM (1956). Ascaroid nematodes from Canadian birds. Can J Zool.

[CR21] Mawson PM, Angel M, Edmonds SJ (1986). A checklist of helminths from Australian birds. Rec S Aust Museum.

[CR22] Mohammad ZA-A, Hbaiel MK (2019) Morphological and molecular study of *Contracaecum* larvae with a new record of *Contracaecum septentrionale* in Al-Sanaf Marsh Southern Thi-Qar Province, Iraq. Ind J Pub Health Res Dev 10(10)

[CR23] Nadler SA, D'Amelio S, Fagerholm HP, Berland B, Paggi L (2000). Phylogenetic relationships among species of *Contracaecum* Railliet & Henry, 1912 and *Phocascaris* Host, 1932 (Nematoda : Ascaridoidea) based on nuclear rDNA sequence data. Parasitology.

[CR24] Roca-Geronès X, Fisa R, Montoliu I, Casadevall M, Tobella C, Bas JM, Palomba M, Mattiucci S (2023). Genetic diversity of *Contracaecum*
*rudolphii* sp. A (Nematoda: Anisakidae) parasitizing the European Shag *Phalacrocorax*
*aristotelis*
*desmarestii* from the Spanish Mediterranean coast. Front Vet Sci.

[CR25] Shamsi S, Gasser R, Beveridge I, Shabani AA (2008). *Contracaecum*
*pyripapillatum* n. sp. and a description of *C*. *multipapillatum* (von Drasche, 1882) from the Australian pelican *Pelecanus*
*conspicillatus*. Parasitol Res.

[CR26] Shamsi S, Norman R, Gasser R, Beveridge I (2009). Genetic and morphological evidences for the existence of sibling species within *Contracaecum*
*rudolphii* (Hartwich, 1964) (Nematoda: Anisakidae) in Australia. Parasitol Res.

[CR27] Shamsi S, Norman R, Gasser R, Beveridge I (2009). Redescription and genetic characterization of selected *Contracaecum* spp. (Nematoda: Anisakidae) from various hosts in Australia. Parasitol Res.

[CR28] Shamsi S, Gasser RB, Beveridge I (2011) Mutation scanning-coupled sequencing of nuclear ribosomal DNA spacers (as a taxonomic tool) for the specific identification of different *Contracaecum* (Nematoda: Anisakidae) larval types. Mol Cellu Probes 25:13–18 10.1016/j.mcp.2010.09.003 20933594

[CR29] Shamsi S, Ghadam M, Suthar J, Ebrahimzadeh Mousavi H, Soltani M, Mirzargar S (2016). Occurrence of ascaridoid nematodes in selected edible fish from the Persian Gulf and description of *Hysterothylacium* larval type XV and *Hysterothylacium*
*persicum* n. sp. (Nematoda: Raphidascarididae). Int J Food Microbiol.

[CR30] Shamsi S, Turner A, Wassens S (2017) Description and genetic characterization of a new *Contracaecum* larval type (Nematoda: Anisakidae) from Australia. J Helminthol 92(2):216–222 10.1017/S0022149X17000360 28473011

[CR31] Shamsi S, Steller E, Chen Y (2018). New and known zoonotic nematode larvae within selected fish species from Queensland waters in Australia. Int J Food Microbiol.

[CR32] Shamsi S, Barton DP, Zhu X (2019). Description and characterisation of *Terranova*
*pectinolabiata* n. sp. (Nematoda: Anisakidae) in great hammerhead shark, *Sphyrna*
*mokarran* (Rüppell, 1837), in Australia. Parasitol Res.

[CR33] Shamsi S, Stoddart A, Smales L, Wassens S (2019b) Occurrence of *Contracaecum bancrofti* larvae in fish in the Murray–Darling Basin. J Helminthol 93(5):574–579. 10.1017/S0022149X1800055X 10.1017/S0022149X1800055X 30017012

[CR34] Zhu X, D'Amelio S, Paggi L, Gasser RB (2000). Assessing sequence variation in the internal transcribed spacers of ribosomal DNA within and among members of the *Contracaecum osculatum* complex (Nematoda: Ascaridoidea: Anisakidae). Parasitol Res.

[CR35] Zhu X, Gasser RB, Jacobs DE, Hung G, Chilton NB (2000). Relationships among some ascaridoid nematodes based on ribosomal DNA sequence data. Parasitol Res.

